# Intonation processing deficits of emotional words among Mandarin Chinese speakers with congenital amusia: an ERP study

**DOI:** 10.3389/fpsyg.2015.00385

**Published:** 2015-04-09

**Authors:** Xuejing Lu, Hao Tam Ho, Fang Liu, Daxing Wu, William F. Thompson

**Affiliations:** ^1^Department of Psychology, Macquarie UniversitySydney, NSW, Australia; ^2^Medical Psychological Institute, The Second Xiangya Hospital, Central South UniversityChangsha, China; ^3^Department of Speech, Hearing and Phonetic Sciences, University College LondonLondon, UK

**Keywords:** congenital amusia, intonation processing, pitch perception, conflict processing, ERP

## Abstract

**Background:** Congenital amusia is a disorder that is known to affect the processing of musical pitch. Although individuals with amusia rarely show language deficits in daily life, a number of findings point to possible impairments in speech prosody that amusic individuals may compensate for by drawing on linguistic information. Using EEG, we investigated (1) whether the processing of speech prosody is impaired in amusia and (2) whether emotional linguistic information can compensate for this impairment.

**Method:** Twenty Chinese amusics and 22 matched controls were presented pairs of emotional words spoken with either statement or question intonation while their EEG was recorded. Their task was to judge whether the intonations were the same.

**Results:** Amusics exhibited impaired performance on the intonation-matching task for emotional linguistic information, as their performance was significantly worse than that of controls. EEG results showed a reduced N2 response to incongruent intonation pairs in amusics compared with controls, which likely reflects impaired conflict processing in amusia. However, our EEG results also indicated that amusics were intact in early sensory auditory processing, as revealed by a comparable N1 modulation in both groups.

**Conclusion:** We propose that the impairment in discriminating speech intonation observed among amusic individuals may arise from an inability to access information extracted at early processing stages. This, in turn, could reflect a disconnection between low-level and high-level processing.

## Introduction

Congenital amusia is a disorder that impacts individuals' ability to discriminate musical pitch. This impairment cannot be explained by hearing or neurological problems, low intelligence, or lack of exposure to music (Ayotte et al., 2002). Instead, it has been linked to a neurodevelopmental failure that renders amusic individuals unable to form stable mental representations of pitch (Patel, 2003, 2008). An important question is whether the pitch deficit accompanying congenital amusia is specific to music or extends to speech perception. Though individuals with amusia rarely report language problems in everyday life (Jiang et al., 2010; Liu et al., 2010) and show normal intonation processing when pitch contrasts are large (Ayotte et al., 2002; Peretz et al., 2002), evidence suggests that amusia does have an effect on individuals' language abilities to some degree. For example, studies have shown that amusics exhibit deficits in processing of lexical tone (Nan et al., 2010; Liu et al., 2012). Additionally, they are reported to have difficulties processing linguistic and emotional prosody in speech (Patel et al., 2008; Jiang et al., 2010; Liu et al., 2010; Thompson et al., 2012).

Speech prosody refers to the meaningful and sometimes paralinguistic acoustic attributes of speech, including pitch, timing, timbre, and intensity. Intonation—the pitch contour of a spoken utterance or “tone of voice”—is one aspect of speech prosody (Selkirk, 1995). When intonation is used to make linguistic distinctions such as the distinction between a question and a statement, it is also referred to as linguistic pitch. The finding that amusic individuals are impaired at processing linguistic pitch suggests that pitch processing is a domain-general function that is engaged when perceiving both music and speech. This possibility aligns with results from studies showing that musical training can lead to enhanced performance on speech perception tasks, including phonological processing (Anvari et al., 2002), speech prosody perception (Thompson et al., 2004; see also Musacchia et al., 2007), linguistic pitch encoding (Wong et al., 2007), and lexical tone identification (Lee and Hung, 2008). It has been argued that such positive “transfer effects” are possible because the brain networks involved in speech and music processing overlap (Patel, 2011).

The ability to process speech prosody is important in daily human communication. Not only does prosody convey linguistic information, it enables listeners to infer a speaker's emotional state. Thompson et al. (2012) found that individuals with amusia exhibit reduced sensitivity to emotional prosody (e.g., happy, sad, and irritated). Nonetheless, such deficits in intonation processing and emotional prosody recognition may not pose a significant problem for amusic individuals when contextual, facial, and linguistic cues are available. As such, impairments to speech perception exhibited by amusic individuals that have been observed in laboratory conditions may disappear in naturalized settings. Indeed, Ayotte et al. (2002) observed that amusic participants were able to discriminate spoken sentences with statement and question intonation, yet showed difficulties processing non-speech analogs in which all linguistic information was filtered out (see also Patel et al., 2005; Hutchins et al., 2010). One interpretation of this finding is that without linguistic information, prosodic information is processed via the (compromised) music mode, resulting in reduced sensitivity; in contrast, the presence of linguistic information might encourage processing via an intact speech mode, preserving sensitivity to speech prosody. It is unclear, however, whether the content of that linguistic information is relevant to this effect. In view of these findings, we examined whether explicit emotional (semantic) cues influence the ability of individuals with amusia to detect subtle pitch changes in speech.

Emotional linguistic information has been shown to facilitate stimulus processing. For example, in the so-called “emotional Stroop” task, in which perceivers are required to name the color of an emotional versus a non-emotional printed word, the former usually gives rise to faster reaction times than the latter (for a review see, e.g., Williams et al., 1996). These results align with a number of findings showing that affective stimuli, such as facial expressions and dangerous animals (e.g., snakes, spiders, etc.), speed up reaction times in visual search tasks (e.g., Fox et al., 2000). Emotional information is generally thought to “grab” perceivers' attention, leading to greater allocation of resources to the stimulus, which, in turn, leads to deeper stimulus processing (for reviews see Compton, 2003; Vuilleumier, 2005). Although some evidence suggests that negative emotional information leads to greater behavioral facilitation than positive emotional information (e.g., Hansen and Hansen, 1988; Öhman et al., 2001; for a review on “negative bias” see Rozin and Royzman, 2001), other evidence indicates that positive stimuli (e.g., “kiss”) can improve performance as effectively as negative stimuli (e.g., “terror”) in tasks, such as the “flanker” and “Simon task” (e.g., Kanske and Kotz, 2010, 2011a,b,c).

The Stroop, Simon, and flanker tasks all induce a response conflict which typically elicits a negative-going ERP component, namely the N2, that peaks between 200 and 350 ms after stimulus onset (for a review see Folstein and Van Petten, 2008). This component has also been shown to be elicited by conflicts between stimulus representations (Yeung et al., 2004). Source localization of the N2 points to neural generators within the anterior cingulate cortex (ACC; Van Veen and Carter, 2002), an area that has been implicated in “conflict monitoring” (Carter, 1998; Botvinick et al., 1999, 2004). In addition to faster reaction times, Kanske and Kotz (2011a,b) observed a conflict-related negativity peaking around 230 ms after stimulus onset that was enhanced for both positive and negative words when compared with neutral words. The time window and characteristic of this conflict-related negativity resembles closely that of the N2.

Findings by Peretz et al. (2005) indicate that brain activity within the N2 time window appears to be impaired in amusia. More specifically, amusics showed a normal N2 response to unexpected small pitch changes (e.g., 25 cents), but they “overreacted” to large pitch changes (e.g., 200 cents) by eliciting an abnormally enlarged N2 when compared to control participants. Nonetheless, Peretz et al. (2005) interpreted amusics' ability to track the quarter-tone pitch difference as indicative of functional neural circuitry underlying implicit perception of fine-grained pitch differences. The observed pitch impairment in amusics arises, according to Peretz et al. (2005, 2009), at a later, explicit stage of processing, as suggested by a larger P3 (Peretz et al., 2005) and the absence of P600 (Peretz et al., 2009) in response to pitch changes in amusics in comparison with controls.

This view has received further support from studies showing normal auditory N1 responses to pitch changes in amusics (Peretz et al., 2005; Moreau et al., 2009). The N1 is a negative-going ERP component that arises between 50 and 150 ms after stimulus onset (e.g., Näätänen and Picton, 1987; Giard et al., 1994; Woods, 1995). Its neural generators have been localized within the auditory cortex (Näätänen and Picton, 1987), suggesting that this component reflects relatively early auditory processing. In contrast to the earlier findings on N1 responses, recent results by Jiang et al. (2012) and Albouy et al. (2013) indicate that pitch processing in amusics may indeed be impaired at early stages of processing, in that the N1 amplitude was significantly smaller for amusics than controls during intonation comprehension (Jiang et al., 2012) and melodic processing (Albouy et al., 2013). Impairments at such an early stage may have consequences for subsequent processes. However, it is unclear whether the pitch deficit exhibited by amusics may be compensated for with linguistic (semantic) cues, where processing takes place relatively late (i.e., ~300–400 ms; for reviews see Pylkkänen and Marantz, 2003; Kutas and Federmeier, 2011). However, findings from ERP research suggest that the emotional content of a (visually presented) word is accessed very early, within 100–200 ms after stimulus onset (e.g., Ortigue et al., 2004; Scott et al., 2009; Palazova et al., 2011; Kissler and Herbert, 2013). Such early processing is thought to be possible via a fast subcortical (thalamao-amygadala) pathway (Morris et al., 1999). Therefore, the early access of emotional semantic information and its facilitative effect on conflict processing could help amusic perceivers overcome any difficulty in discriminating linguistic pitch.

To address this question, we presented emotional words spoken with intonation that indicated either a statement or a question, and recorded EEG responses in individuals with and without amusia. The linguistic content of the words had either a positive valence, such as “joy,” or a negative valence, such as “ugly.” The task was to judge whether two successively presented words were the same in intonation. If amusics make use of linguistic information to compensate for any impairment in intonation processing, they should perform as well as control participants on the intonation-matching task. However, emotional semantic cues may be insufficient to facilitate subsequent processing in amusic individuals. In this case, we would expect to see differences in brain activity between amusic and control participants within an early time window, such as that of the N1 component. Alternatively, early, implicit auditory processes may be intact in amusics and the observed pitch impairment may arise only at a later, explicit processing stage (e.g., N2). In this case, amusic participants should show comparable brain activity to normal controls within the early but not late time window.

## Materials and methods

### Participants

Twenty individuals with congenital amusia (17 females; age: *M* = 21.85 years, *SD* = 2.11 years; year of education: *M* = 15.25 years, *SD* = 2.10 years) and 22 matched control participants (16 females; age: *M* = 20.68 years, *SD* = 1.81 years; year of education: *M* = 14.32 years, *SD* = 1.25 years) were tested. All participants were Mandarin native speakers and right-handed. None reported any auditory, neurological, or psychiatric disorder. No one had taken private music lessons or other extracurricular music training beyond basic music education at school. All participants gave written informed consent prior to the study. The Ethics Committee of the Second Xiangya Hospital approved the experimental protocol. Participants with a mean global percentage correct lower than 71.7% in the Montreal Battery of Evaluation of Amusia (MBEA; Peretz et al., 2003) were classified as amusic, corresponding to 2SD below the mean score of the Chinese norms (Nan et al., 2010). The MBEA consists of three melodic pitch-based tests (Scale, Contour and Interval), two time-based tests (Rhythm and Meter) and one memory test (Memory). For the first four subtests, listeners are presented with pairs of melodies and asked to judge whether they are the “same” or “different.” For the last two subtests, listeners are presented with a single melody on each trial. For the Meter subtest, participants are required to judge whether the presented melody is a “March” or a “Waltz.” In the Memory subtest, participants are required to judge whether they have heard the presented melody in the preceding subtests. The results of the MBEA and its subtests for both groups are shown in Table 1.

**Table 1:** **Participants' mean proportion correct responses (standard deviations in parentheses) and independent-samples *t*-tests results on the MBEA and its subtests between amusic and control groups**.

	**Amusics (*n* = 20)**	**Controls (*n* = 22)**	***t*-value**	***p*-value (2-tailed)**	**Cohen's *d***
Scale	0.63 (0.08)	0.92 (0.06)	13.42	<0.01	4.13
Contour	0.66 (0.09)	0.93 (0.07)	10.92	<0.01	3.37
Interval	0.59 (0.06)	0.89 (0.07)	15.79	<0.01	4.58
Rhythm	0.71 (0.11)	0.91 (0.07)	7.36	<0.01	2.19
Meter	0.64 (0.17)	0.85 (0.15)	4.19	<0.01	1.31
Memory	0.72 (0.11)	0.96 (0.04)	8.88	<0.01	2.96
Global score	0.66 (0.03)	0.91 (0.04)	22.66	<0.01	7.02

*Individuals with amusia scored significantly lower than control participants on all subtests of the MBEA (ps < 0.01)*.

### Stimuli

**Figure 1:**
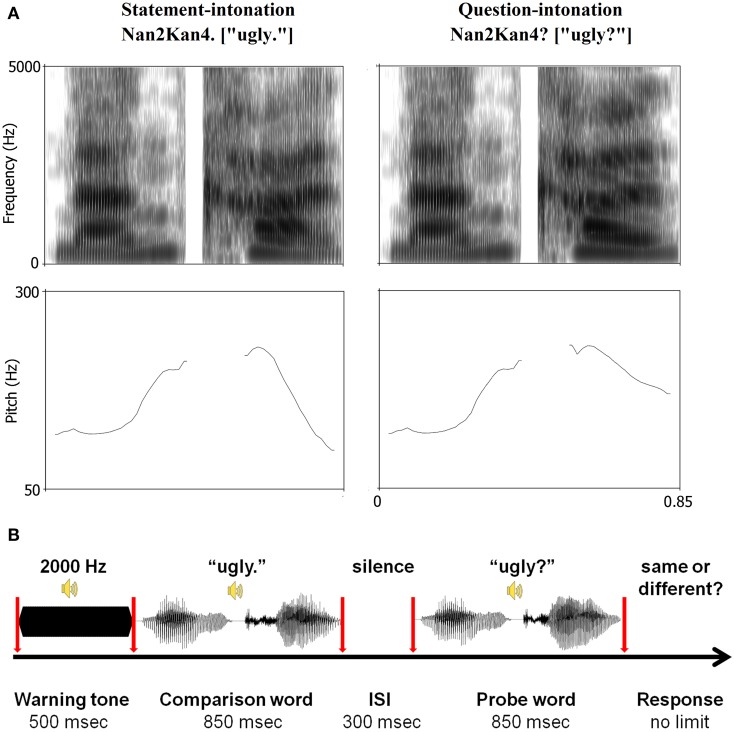
**(A)** Spectrogram and pitch contours of a pair of stimuli used in the task and **(B)** the scheme of trial timeline. The negative word “
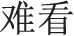
” (nan2kan4), which means “ugly,” as a statement (left panel) and a question (right panel). The mean F_0_ of statement- and question-intonation was identical in terms of the first syllable (positive words: *M* = 179.74 Hz, *SD* = 39.23 Hz; negative words: *M* = 194.35 Hz, *SD* = 80.56 Hz), but it was different in terms of the second syllable (positive statement: *M* = 170.57 Hz, *SD* = 62.76 Hz; positive question: *M* = 223.88 Hz, *SD* = 57.51 Hz; negative statement: *M* = 199.89 Hz, *SD* = 80.69 Hz; negative question: *M* = 226.64 Hz, *SD* = 64.77 Hz). All trials started with a 2000 Hz sinusoidal lasting for 500 ms. After the presentation of the comparison word (two 400 ms syllables with a 50 ms silence between them), a 300 ms silence was presented, followed by the probe word lasting for 850 ms. During the task, participants were asked to fixate on a white cross on a black screen. At the end of each trial, they were required to make a non-speeded response to indicate whether the intonation of the comparison and probe words was the same or different by pressing one of two response keys.

The stimulus material consisted of a set of 40 disyllabic words from the Chinese Affective Words Categorize System (CAWCS; Xu et al., 2008), which comprises 230 positive (e.g., “joy,” “happy,” and “excited”) and negative (e.g., “ugly,” “depressed,” and “poor”) words. All words from the CAWCS were recorded by an adult male Mandarin native speaker who spoke each word as a statement and as a question. Seven Mandarin native speakers (5 females) were asked to rate on a five-point scale how well the intonations were recognized as a statement or a question (1 = definitely a statement, 5 = definitely a question). Twenty positive and twenty negative words, whose rating scores were equal to or lower than 2 in statement-intonation and equal to or higher than 3.5 in question-intonation were selected. This corresponds approximately to the 30 and 70 percentiles of the ratings respectively. Independent-samples *t*-tests confirmed that the selected negative and positive words yielded similar mean rating scores in both statement and question conditions (*ps* > 0.35, see Table 2). Additional one-sample *t*-tests indicated that the mean valence, arousal, and familiarity scores for the 40 selected words were not significantly different than that of the 230 words from the CAWCS (*ps* > 0.1). However, a comparison of the selected positive and negative words revealed that the former were rated as more arousing and more familiar than the latter (*ps* < 0.01, see Table 3)^1^ Using a cross-splicing technique (for more details see Patel et al., 1998), we ensured that the first syllables were acoustically identical and the durations of the second syllables were roughly equal. Figure 1A shows the spectrogram and pitch contours of a negative word spoken with a statement-intonation and a question-intonation. As in Jiang et al. (2010), each word was set to be 850 ms, that is, each syllable lasted 400 ms and there was a 50 ms silence between the two syllables.

**Table 2:** **The mean intonation rating of the selected words across 7 raters (standard deviations in parentheses) and the independent-samples *t*-tests results comparing positive and negative words**.

	**Positive words**	**Negative words**	***t*-value**	***p*-value (2-tailed)**	**Cohen's *d***
Statement	1.25 (0.21)	1.19 (0.18)	0.94	0.35	0.31
Question	4.18 (0.24)	4.19 (0.25)	0.18	0.86	0.04

**Table 3:** **The mean rating of valence, arousal and familiarity of the selected words (standard deviations in parentheses) and the independent-samples *t*-tests results on each dimension comparing positive and negative words**.

	**Positive words**	**Negative words**	***t*-value**	***p*-value (2-tailed)**	**Cohen's *d***
Valence	7.28 (0.57)	2.72 (0.30)	31.57	<0.01	10.01
Arousal	6.54 (0.50)	5.42 (0.42)	7.66	<0.01	2.43
Familiarity	6.06 (0.66)	4.55 (0.52)	8.06	<0.01	2.54

*CAWCS employed eight-point scale (1 = lowest, 8 = highest) to evaluate the valence, arousal, and familiarity of each word*.

### Procedure

Participants were seated in an electrically shielded and sound-attenuated room with dimmed light. They were asked to fixate on a white cross on a black CRT monitor screen. As illustrated in Figure 1B, each trial began with a warning tone (2000 Hz sinusoidal) of 500 ms. Subsequently, a comparison word was presented, followed by an inter-stimulus interval (ISI) of 300 ms. Thereafter, participants heard the probe word. They were asked to judge whether the intonation of the probe word was the same as that of the comparison word by pressing one of two response keys. The auditory stimuli were presented binaurally at a comfortable listening level via earphones.

### Experimental design

The experiment consisted of 2 blocks separated by a break. All trials were presented in a pseudo-randomized order. Each block consisted of 80 congruent or incongruent trials (20 statement-statement pairs, 20 statement-question pairs, 20 question-question pairs, and 20 question-statement pairs). Prior to the testing, participants completed 4 practice trials to familiarize themselves with the stimuli and task. Feedback was provided in the practice but not the experimental trials. For stimulus presentation and data collection, we employed the software *Stim2* (Compumedics Neuroscan, USA).

### EEG recording and pre-processing

The EEG was recorded from 32 electrodes *Quick-cap* (standard 10–10 electrode system) with a *SynAmps RT* amplifier and the *SCAN* software from NeuroScan System (Compumedics Neuroscan, USA). The average of the left and right mastoids served as the reference during recording. Vertical and horizontal eye movements and blinks were monitored with 4 additional electrodes. All electrode impedances were kept below 5 kΩ during the experiment. An online bandpass filter of 0.05–50 Hz was used during the recording. The sampling rate was 500 Hz.

The EEG was processed in *MATLAB* (Version R2013b; MathWorks, USA) using the *EEGLAB* toolbox (Delorme and Makeig, 2004). The data were first highpass filtered with a Windowed Sinc FIR Filter (Widmann and Schröger, 2012) from the EEGLAB plugin *firfilt* (Version 1.5.3). The cutoff frequency was 2 Hz (Blackman window; filter order: 2750). An independent component analysis (ICA) was performed using the *runica* algorithm. Subsequently, an ICA based method for identifying ocular artifacts, such as eye movements and blinks were used (Mognon et al., 2011). Artifactual components were rejected and a lowpass Windowed Sinc FIR Filter with a 20 Hz cutoff frequency (Blackman window; filter order: 138) was applied. Epochs of -500 to 1450 ms from the onset of probe words were extracted and baseline corrected using the 500 ms pre-stimulus time period.

### ERP data analyses

Visual inspection of the grand averages revealed two pronounced negative ERP deflections in the following time windows: 120–180 ms and 250–320 ms after the onset of the second syllable of the probe word. These negativities likely reflect the N1 and N2 components, which typically peak within similar time windows (e.g., Pérez et al., 2008; Peretz et al., 2009; Astheimer and Sanders, 2011). For statistical analysis, except for four outer scalp electrodes (T7, T8, O1, O2), all other electrodes were grouped into four regions of interest (ROI): left-anterior (FP1, F3, FC3, F7, FT7), right-anterior (FP1, F4, FC4, F8, FT8), left-posterior (C3, CP3, P3, P7, TP7), and right-posterior (C4, CP4, P4, P8, TP8). The midline electrodes were analyzed separately and grouped into mid-anterior (FZ, FCZ, CZ) and mid-posterior electrodes (CPZ, PZ, OZ). Mean amplitudes were computed for each region of interest and time window (Luck, 2005). Separate repeated-measures ANOVAs were conducted on the N1 and N2 time windows. The factors entered into the ANOVAs were: Group (control/amusic), Emotion (positive/negative), Congruence (congruent/incongruent intonation), LR (left/right), and AP (anterior/posterior). The factor LR was excluded from the analyses of the midline electrodes. The statistical results for the N1 and N2 time window are summarized respectively (see Supplementary Material). Partial *eta squared* and cohen's *d* were used to evaluate the effect size for the ANOVAs and *t*-tests, respectively. Below, we will only report in detail main effects and interactions of interest (see the Supplementary Tables 1 and 2 for full results).

## Results

### Task performance

Participants' task performance was evaluated using d-prime (d')—a measure of discriminability or sensitivity (Macmillan and Creelman, 2005). D-prime scores were calculated by subtracting the z-score that corresponds to the false-alarm rate from the z-score that corresponds to the hit rate. A standard correction was applied to hit and false-alarm rates of 0 or 1 by replacing them with 0.5/n and (n-0.5)/n, respectively, where n is the number of incongruent or congruent trials (Macmillan and Kaplan, 1985). A repeated-measures ANOVA was conducted on the d' scores with two factors: Group (control/amusic) and Emotion (positive/negative). The results revealed a significant main effect of Group, *F*_(1, 40)_ = 11.05, *p* < 0.01, η^2^ = 0.22, but no significant main effect of Emotion, *F*_(1, 40)_ = 0.02, *p* > 0.90, η^2^ < 0.01, nor a significant interaction between Emotion and Group, *F*_(1, 40)_ = 0.85, *p* >0.36, η^2^ = 0.02. Inspection of the means revealed that individuals with amusia (positive words: *M* = 1.56, *SD* = 0.94; negative words: *M* = 1.63, *SD* = 1.01) made more errors than controls (positive words: *M* = 2.40, *SD* = 0.57; negative words: *M* = 2.34, *SD* = 0.56) in the matching task.

### EEG results

#### N1 (120–180 ms) time window

**Figure 2:**
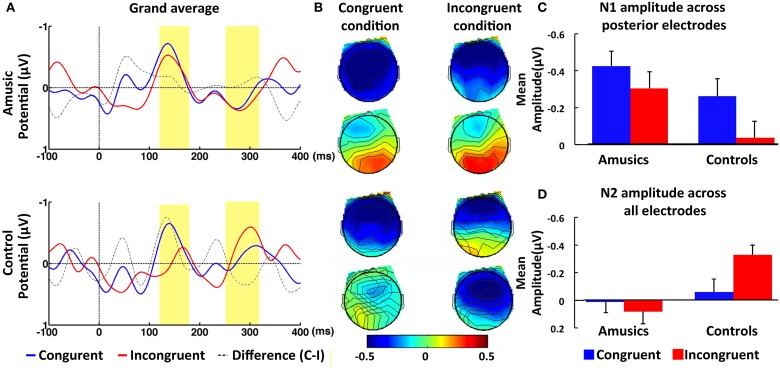
**ERP results in response to congruent and incongruent intonations within N1 time window (120–180 ms) and N2 time window (250–320 ms). (A)** Grand-averaged ERPs at posterior electrode CP4 in response to congruent (blue line) and incongruent intonation (red line) for amusic (upper panel) and for control (lower panel) participants. The time windows of the N1 and N2 were highlighted (in yellow). **(B)** Topographic maps of average amplitude (μV) in N1 and N2 time window averaged over all electrodes for amusics (upper panel) and for controls (lower panel). **(C)** Mean amplitude averaged over the ROI of posterior electrode sites within N1 time window for congruent trials (blue bar) and incongruent trials (red bar). **(D)** Mean amplitude averaged over the ROI of all electrode sites within N2 time window for congruent trials (blue bar) and incongruent trials (red bar). Error bars represent 1 SEM.

**Figure 3:**
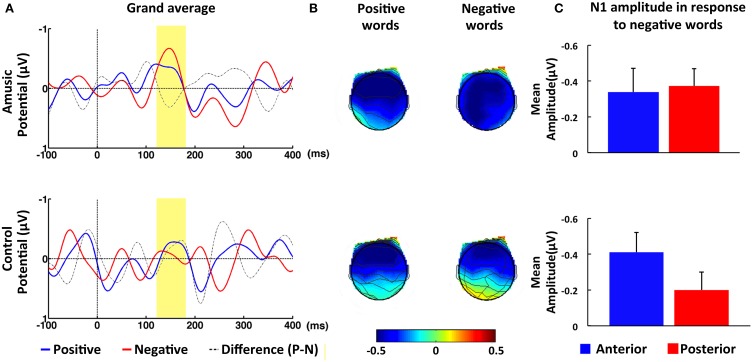
**ERP results in response to positive and negative words within N1 time window (120–180 ms). (A)** Grand-averaged ERPs at posterior electrode PZ in response to positive (blue line) and negative words (red line) for amusic (upper panel) and for control (lower panel) participants. The time window of the N1 was highlighted (in yellow). **(B)** Topographic maps of average amplitude (μV) in N1 time window averaged over all electrodes. **(C)** Mean amplitude averaged over the ROI of anterior (blue bar) and posterior (red bar) electrode sites in response to negative words within N1 time window. Error bars represent 1 SEM.

Individuals with amusia showed an N1 amplitude comparable to that of normal controls, as confirmed by the non-significant main effect of Group, *F*_(1, 40)_ = 0.68, *p* = 0.42, η^2^ = 0.02. Furthermore, a significant Congruence × AP interaction was observed, *F*_(1, 40)_ = 5.23, *p* < 0.05, η^2^ = 0.12 Similar to the control participants, amusia participants displayed a reduced N1 amplitude at posterior electrodes in response to incongruent, *M* = −0.26, *SE* = 0.10, than congruent intonation, *M* = −0.43, *SE* = 0.08. This congruence effect appears to be constrained to the posterior electrodes, as paired-sample *t*-tests yielded a significant difference between the congruent and incongruent condition only at posterior, *t*_(41)_ = 2.70, *p* < 0.01, *d* = 0.42, but not anterior electrode sites, *t*_(41)_ = 0.24, *p* > 0.81, *d* = 0.04 (see Figure 2). Additionally, the ANOVA revealed a significant interaction involving Emotion, Group, LR, and AP, *F*_(1, 40)_ = 5.49, *p* < 0.05, η ^2^ = 0.12 When this complex interaction was unpacked by Emotion, we found a significant interaction between Group and AP for negative words, *F*_(1, 40)_ = 4.88, *p* < 0.05, η^2^ = 0.11, but not for positive words, *F*_(1, 40)_ = 0.06, *p* > 0.81, η^2^ < 0.01. Further analyses with paired-sample *t*-tests revealed that normal controls showed a significantly larger N1 response, *t*_(21)_ = 3.88, *p* < 0.01, *d* = 0.83, to negative words at anterior, *M* = −0.42, *SE* = 0.11, than posterior electrode sites, *M* = −0.14, *SE* = 0.11. No such topographical difference was found in the amusic group, *t*_(19)_ = 0.17, *p* > 0.86, *d* = 0.04, suggesting that the N1 was broadly distributed in this group (see Figure 3). A direct comparison between the amusic and control group using an independent-samples *t*-test revealed a larger N1 response to negative words for amusic participants, *M* = −0.40, *SE* = 0.09, as compared to control participants, *M* = −0.14, *SE* = 0.11, at posterior electrode sites; however, this difference was only marginally significant, *t*_(40)_ = 1.88, *p* = 0.07, *d* = 0.29. At anterior electrode sites, the *t*-test yielded no significant difference (*p* > 0.97).

#### N2 (250–320 ms) time window

**Figure 4:**
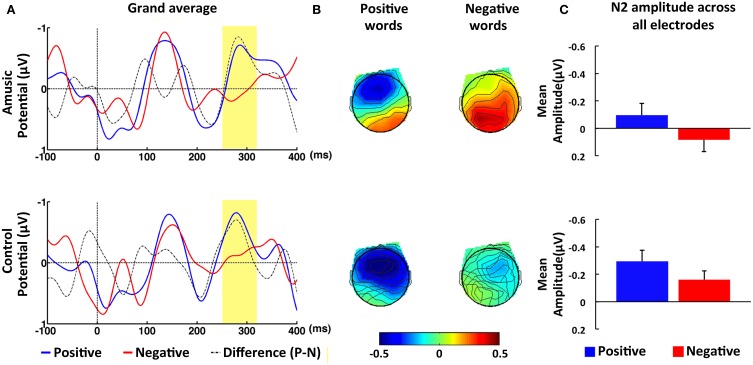
**ERP results in response to positive and negative words within N2 time window (250–320 ms). (A)** Grand-averaged ERPs at fronto-central electrode FZ in response to positive (blue line) and negative words (red line) for amusic (upper panel) and for control (lower panel) participants. The time window of the N2 was highlighted (in yellow). **(B)** Topographic maps of average amplitude (μV) in N2 time window averaged over all electrodes. **(C)** Mean amplitude averaged over the ROI of all electrode sites within N2 time window for positive (blue bar) and negative words (red bar). Error bars represent 1 SEM.

In contrast to the N1 time window, amusic participants showed different ERPs in comparison to control participants in response to congruent and incongruent intonations within the N2 time window (see Figure 2). This was confirmed by the repeated-measures ANOVAs, which yielded a significant Group difference in the ROIs as well as at the midline electrodes, *F*_(1, 40)_ = 6.35, *p* < 0.05, η^2^ = 0.14, and *F*_(1, 40)_ = 6.43, *p* < 0.05, η^2^ = 0.14, respectively. In addition, Group factor showed a marginally significant interaction with Congruence factor, *F*_(1, 40)_ = 3.87, *p* < 0.06, η^2^ = 0.09, which was further analyzed with two independent-sample *t*-tests. The results revealed that amusic participants elicited a smaller N2 amplitude, *M* = 0.08, *SE* = 0.09, than control participants, *M* = −0.32, *SE* = 0.08, in the incongruent condition, *t*_(40)_ = 3.28, *p* < 0.01, *d* = 0.51. No such difference was found in the congruent condition, *t*_(40)_ = 0.55, *p* > 0.58, *d* = 0.08. It should be noted, however, that although visual inspection indicated that control participants exhibited a large N2 response to incongruent in comparison to congruent probe words, *M* = −0.05, *SE* = 0.10, this difference was only marginally significant when probed with a paired-sample *t*-test, *t*_(21)_ = 1.92, *p* = 0.07, *d* = 0.41. Amusic participants showed no such trend toward the congruence effect, *t*_(19)_ = 0.73, *p* > 0.47, *d* = 0.16. Finally, the results included a significant main effect of Emotion in the ROIs, *F*_(1, 40)_ = 17.63, *p* < 0.01, η^2^ = 0.31, as well as at the midline electrodes, *F*_(1, 40)_ = 11.60, *p* < 0.01, η^2^ = 0.23 The means computed across the ROIs point to a larger N2 amplitude for positive, *M* = −0.20, *SE* = 0.06, as compared to negative words, *M* = −0.04, *SE* = 0.06. Emotion and Group did not interact significantly in the four ROIs, *F*_(1, 40)_ = 0.34, *p* = 0.56, η^2^ = 0.01, suggesting that amusic and control participants showed a similar effect of Emotion (see Figure 4).

In summary, our main findings showed that amusic participants made more errors compared with control participants in the intonation matching task, despite the emotional content of the words presented. In terms of brain activities, both groups exhibited similar N1 response to the conflicting intonations as hypothesized (Peretz et al., 2005; Moreau et al., 2009). However, the N1 elicited by negative words was marginally larger in amusics than in controls at posterior electrode sites. Finally, when compared to controls, amusics showed a significantly reduced N2 amplitude in response to incongruent intonation.

## Discussion

The present study investigated three related questions. First, do individuals with congenital amusia show impairment in processing speech prosody? Second, can amusic participants make use of emotional information to compensate for any impairment in speech prosody processing? Third, does the impairment in pitch processing in amusia arise from an early or late stage of processing? To address these questions, we measured the brain activities of participants with and without congenital amusia using EEG. Participants were presented with pairs of positive (e.g., “joy”) or negative spoken words (e.g., “ugly”) successively. The pairs were congruent or incongruent in terms of speech intonation, which could indicate a statement or a question. Participants were asked to indicate whether the word pairs had the same or different intonation.

As speakers of a tone language, Mandarin Chinese amusics may be sensitive to linguistic pitch owing to constant exposure to daily communication with small changes in pitch (for a discussion, see Stewart and Walsh, 2002; Stewart, 2006). However, the present results indicate that amusic participants had difficulty discriminating between statements and questions. This finding is consistent with other evidence that Mandarin amusics exhibit mild deficits in intonation identification and discrimination in comparison with controls (Jiang et al., 2010). More generally, the failure in linguistic pitch discrimination among tone language speakers with amusia challenges the view that amusia is a disorder specific to musical pitch perception (Ayotte et al., 2002; Peretz et al., 2002), as the musical pitch impairment extended to the domain of language (see also, Patel et al., 2008; Nguyen et al., 2009; Jiang et al., 2010; Liu et al., 2010; Nan et al., 2010; Tillmann et al., 2011). It should be emphasized, however, that there is considerable debate concerning the degree to which musical pitch impairment negatively impacts upon linguistic pitch perception. A number of studies have shown that linguistic pitch discrimination is significantly worse among amusics when semantic information is artificially removed (i.e., when only prosody is presented in non-speech analogs) than when natural speech is presented (e.g., Ayotte et al., 2002; Patel et al., 2005). This finding implies that amusic individuals can make use of semantic cues to compensate for their pitch deficit, as shown in Liu et al. (2012). In the present study, participants were provided with emotional semantic cues and were asked to match the intonation of negatively or positively valenced words. In order to perform this task successfully, the participants needed to be able to detect the conflict in intonations of comparison and probe words. Although it has been suggested that both positive and negative words can ease conflict processing (Kanske and Kotz, 2010, 2011a,b, c), thereby facilitating behavioral performance, our behavioral results revealed that the impairment of linguistic intonation discrimination among amusic individuals was still observed when intonation was applied to words with positive or negative emotional valence. This finding suggests that emotional valence failed to facilitate pitch processing in individuals with amusia.

Correspondingly, we found the N2 elicited in conflict trials to be significantly reduced in amusics as compared with controls. As the amplitude of the N2 is typically larger in conflict than non-conflict trials (Nieuwenhuis et al., 2003), this finding further suggests that conflict processing was virtually absent in the amusic group. On the other hand, our ERP results revealed no impaired emotion processing in amusic individuals. Both amusic and control groups exhibited a larger N2 amplitude for positive words as compared with negative words, which likely reflects the higher arousal level ascribed to the positive than negative words employed in the experiment (see Table 3 and Supplementary Table 2). These findings suggest that amusics' failure to discriminate between question and statement intonation arises from an impairment related to conflict processing, rather than from an inability to process emotional information. The abnormal N2 observed in the amusic group is in part consistent with the results by Peretz et al. (2005) who also reported abnormal brain activity within the N2 time window in amusic as compared with control participants. However, in contrast to the present study, Peretz et al. (2005) employed an oddball paradigm and found that the amusic brain “overreacted” to unexpected (infrequent) pitch changes by eliciting a larger N2 response than normal controls. Internally generated expectancy caused by stimulus probability has been shown to contribute to the N2 response (see Folstein and Van Petten, 2008 for a review). Therefore, the greater N2 amplitude in the amusic group observed by Peretz et al. (2005) may partially reflect processes related to expectancy. When, in a later study, the conflicting pitch (an out-of-key note) occurred more frequently and, hence, less unexpectedly, Peretz et al. (2009) observed, similar to the present findings, that controls but not amusics elicited a large N2 response to the conflicting pitch.

Contrary to our results for the N2 response, the reduction in N1 in response to incongruent intonation was similar in amusic and control participants. These results corroborate earlier finding by Jiang et al. (2012), in which participants judged whether aurally-presented discourse was semantically acceptable. The same pattern of N1 in two groups suggested that the underlying process is normal in the amusic group (see also Peretz et al., 2005, 2009; Moreau et al., 2009). However, other studies have reported an abnormal N1 response in amusics during intonation comprehension (Jiang et al., 2012) and melodic processing (Albouy et al., 2013). To reconcile these contradictory findings, Albouy et al. (2013) proposed that whether amusic participants show a normal or abnormal N1 may depend on task difficulty. Studies that reported a normal N1 used tasks that were relatively easy, such as a deviant tone detection task (Peretz et al., 2005, 2009) or no task at all (Moreau et al., 2009). In contrast, Albouy et al. (2013) and Jiang et al. (2012) employed tasks in which participants had to match two melodies, and judge whether a speech intonation was appropriate or inappropriate given a certain discourse, respectively. These authors found the N1 in individuals with amusia to be abnormal. Our behavioral results suggest that the task we used was difficult for the amusic participants (see the above discussion). Yet, we found a normal N1 for the amusic group.

One explanation is that the emotional words used in the present study led to enhanced attention which, in turn, improved pitch processing in amusic participants. This gave rise to a relatively normal N1 response, despite the observed task difficulty in the amusic group. It should be noted that as neutral words were not included in this study, it is not possible to assess whether emotional valence benefited performance behaviorally. Nonetheless, for reasons that we will elucidate below, it is possible there was a small effect of emotional valence that was insufficient to boost amusic participants' task performance to the level of controls. As suggested by our ERP results, amusic participants were affected by negative words differently than normal controls at an early processing stage, i.e., the N1 time window. More specifically, we observed a larger N1 amplitude in the amusic group in comparison to the control group; however, this difference was only marginally significant and restricted to the posterior electrode sites. The auditory N1 has been shown to be modulated by selective attention and to increase in amplitude when perceivers direct their attention to the stimulus (e.g., Woldorff et al., 1993; Alho et al., 1994; for a review see, e.g., Schirmer and Kotz, 2006). Thus, the larger N1 response displayed by the amusic participants could reflect enhanced attention to the negative words.

No significant group difference at either anterior or posterior electrode sites was found in the positive word condition. Negative stimuli have been shown to lead to better performance than positive stimuli (e.g., Hansen and Hansen, 1988; Öhman et al., 2001), which suggests that negative stimuli are more effective in capturing attention than positive stimuli. This has often served as an argument in support of the “negativity bias” hypothesis according to which we may have developed some adaptive mechanisms to deal with negative emotions (for a review see Rozin and Royzman, 2001). It should be noted that when examining the N1 response at anterior and posterior electrode sites within each group, we found in the control group a significantly larger N1 response to negative words at posterior than at anterior electrodes. In contrast, the amusic group showed comparable N1 amplitudes at both electrode sites. The broad scalp distribution of the N1 response displayed by amusic participants could indicate some additional activation of posterior brain areas that were not present in normal participants. Consistent with the notion of enhanced attention in the amusic group, these additional areas may be linked to attentional processes.

In short, our results suggest that amusics may process emotional words (negative valenced in the present study) in a manner that differs from individuals without this impairment, potentially compensating for their disorder. However, this enhanced processing may not have been sufficient to improve the amusic participants' performance. Our failure to find a clear emotion effect in the behavioral and ERP data may be due to the low arousal level of the emotional words we used, e.g., “ugly.” In comparison, Kanske and Kotz (2011c), for instance, used words such as “terror,” which elicited clear emotion effects. This may also explain why we did not observe a “negativity bias” in our control group, as the negative words were lower in arousal when compared with positive words.

To interpret the N1 and N2 results together, we propose that the impairment in discriminating speech intonation observed among amusic individuals may arise from an inability to access information extracted at early processing stages. This inability, in turn, could reflect some disconnection between low-level and high-level processing. Conflict detection is generally thought to play a pivotal role in cognitive control. Following the detection of a conflict, perceivers presumably increase their attention and make “strategic adjustments in cognitive control” (Botvinick et al., 2001, 2004), resulting in reduced interference in subsequent trials (Kerns et al., 2004). Therefore, a deficit in conflict detection can have severe consequences on behavior.

Many of the cognitive and social deficits associated with schizophrenia are believed to arise from impairments in conflict detection and cognitive control (Carter, 1998). Typically, the activation of ACC is only affected by conflicting stimuli perceived consciously but not subliminally in normal perceivers, whereas individuals with schizophrenia exhibit impaired conscious but normal subliminal priming (Dehaene et al., 2003). But the situation for amusia is unlike schizophrenia for whom the ACC is considered to be dysfunctional and the conscious control network is affected (Alain et al., 2002; Kerns et al., 2005). If a conflict in pitch cannot even be detected, amusic perceivers would not have an opportunity to become aware of the conflict, even though at a lower processing level, pitch discrimination is intact, as suggested by our N1 findings.

A recent study reported a similar dissociation between lexical tone identification and brainstem encoding of pitch in speech (Liu et al., 2015), which suggests that high-level linguistic pitch processing deficits in amusia operate independently of low-level brainstem functioning. We can only speculate that access to this low-level information is limited in individuals with amusia. Dehaene et al. (2006) have usefully distinguished “accessibility” from “access,” whereby some attended stimuli have the potential to gain access to conscious awareness (accessibility), but they are nonetheless not consciously accessed (access). Thus, it is possible that pitch information processed at an early stage is potentially accessible, but amusic individuals do not have conscious access to that information.

In conclusion, the present investigation provides further evidence that the pitch deficit associated with congenital amusia extends to the domain of language, corroborating the hypothesis that music and language processing share common mechanisms. Speaking a tone language, such Mandarin Chinese, does not compensate for this deficit. However, in daily life, amusic perceivers may make use of other cues, such as linguistic information, to compensate for their impairment. Our results suggest that individuals with amusia are more sensitive to linguistic emotional information than normal participants and that this sensitivity has some influence on early stages of pitch processing (i.e., in the N1 time window). However, emotional modulations appear to be restricted to this early processing stage. At a later processing stage (i.e., in the N2 time window), amusic participants still exhibit impairments in detecting conflicting intonation. We suggest that this impairment stems from an inability to access information extracted at earlier processing stages (e.g., the N1 time window), reflecting a disconnection between low-level and high-level processing in this population. It should be noted that the effect sizes of the findings here are small, owing to the nature of the linguistic stimuli and a low EEG signal-to-noise ratio (20 trials per condition). Future investigations of these questions may benefit from a larger number of trials in each condition to increase the signal-to-noise ratio.

### Conflict of interest statement

The authors declare that the research was conducted in the absence of any commercial or financial relationships that could be construed as a potential conflict of interest.

## Footnotes

^1^It should be noted that the positive and negative words used here differ in valence, arousal and familiarity (also see Kanske and Kotz, 2010). However, the purpose of this study was to examine the extent to which emotional semantics as a whole are relevant to task performance in an amusic population.
