# Supplementary material for: Intonation processing deficits of emotional words among Mandarin Chinese speakers with congenital amusia: an ERP study
Source: Front Psychol. 2015 Apr 9;6:385. doi: 10.3389/fpsyg.2015.00385 (PMC4391227; doi:10.3389/fpsyg.2015.00385)
Supplement: Supplementary file 1 [file Table1.PDF]

# *Supplementary Material*

# Intonation Processing Deficits of Emotional Words among Mandarin Chinese Speakers with Congenital Amusia: An ERP Study

**Xuejing Lu [1,2], Hao Tam Ho[1], Fang Liu[3], Daxing Wu[2]*, William F. Thompson[1]***

[1] Department of Psychology, Macquarie University, Sydney, NSW, Australia
[2] Medical Psychological Institute, the Second Xiangya Hospital, Central South University, Changsha, China
[3] Department of Speech, Hearing and Phonetic Sciences, University College London, London, UK

*** Correspondence:**

Daxing Wu, Medical Psychological Institute, the Second Xiangya Hospital, Central South University, No.139 Middle Renmin Road, Changsha, 410011, China.
wudaxing2012@126.com

William F. Thompson, Department of Psychology, Macquarie University, NSW 2109, Australia
bill.thompson@mq.edu.au

## Supplementary Tables

**Supplementary Table 1. Statistical results for the N1 (120 - 180 msec) time window.** Summary of the repeated-measures ANOVA on the mean amplitudes computed across the four regions of interest (ROI) and midline electrode sites, with the factors of Group (control / amusic), Emotion (positive / negative), Congruence (congruent / incongruent), LR (left / right) and AP (anterior / posterior). Effect size was estimated using partial eta-squared ($\eta^2$). DF refers to the degrees of freedom and the asterisks (*) indicate effects that yielded a significance level of $p < 0.05$.

| | Effect | DF*n* | DF*d* | F | p | $\eta^2$ | p < 0.05 |
|---|---|---|---|---|---|---|---|
| **ROI** | **Group** | 1 | 40 | 0.68 | 0.42 | 0.02 | |
| | **Emotion** | 1 | 40 | 0.09 | 0.77 | 0.00 | |
| | **Emotion × Group** | 1 | 40 | 0.18 | 0.67 | 0.01 | |
| | **Congruence** | 1 | 40 | 2.52 | 0.12 | 0.06 | |
| | **Congruence × Group** | 1 | 40 | 0.38 | 0.54 | 0.01 | |
| | **LR** | 1 | 40 | 0.11 | 0.75 | 0.00 | |
| | **LR × Group** | 1 | 40 | 0.21 | 0.65 | 0.01 | |

| | | | | | | | |
|---|---|---|---|---|---|---|---|
| | **AP** | 1 | 40 | 19.35 | <0.01 | 0.33 | * |
| | **AP× Group** | 1 | 40 | 2.75 | 0.11 | 0.06 | |
| | **Emotion × Congruence** | 1 | 40 | 0.42 | 0.52 | 0.01 | |
| | **Emotion × Congruence × Group** | 1 | 40 | 0.09 | 0.77 | 0.00 | |
| | **Emotion × LR** | 1 | 40 | 0.00 | 0.99 | 0.00 | |
| | **Emotion × LR × Group** | 1 | 40 | 2.83 | 0.10 | 0.07 | |
| | **Congruence × LR** | 1 | 40 | 0.01 | 0.95 | 0.00 | |
| | **Congruence × LR × Group** | 1 | 40 | 0.40 | 0.53 | 0.01 | |
| | **Emotion × Congruence × LR** | 1 | 40 | 0.05 | 0.83 | 0.00 | |
| | **Emotion × Congruence × LR × Group** | 1 | 40 | 0.31 | 0.58 | 0.01 | |
| | **Emotion × AP** | 1 | 40 | 1.07 | 0.31 | 0.03 | |
| | **Emotion × AP × Group** | 1 | 40 | 1.46 | 0.23 | 0.04 | |
| | **Congruence× AP** | 1 | 40 | 5.23 | 0.03 | 0.12 | * |
| | **Congruence× AP × Group** | 1 | 40 | 0.02 | 0.89 | 0.00 | |
| | **Emotion × Congruence × AP** | 1 | 40 | 1.17 | 0.29 | 0.03 | |
| | **Emotion × Congruence × AP × Group** | 1 | 40 | 0.15 | 0.70 | 0.00 | |
| | **LR × AP** | 1 | 40 | 0.45 | 0.51 | 0.01 | |
| | **LR × AP × Group** | 1 | 40 | 0.44 | 0.51 | 0.01 | |
| | **Emotion × LR × AP** | 1 | 40 | 0.64 | 0.43 | 0.02 | |
| | **Emotion × LR × AP × Group** | 1 | 40 | 5.49 | 0.02 | 0.12 | * |
| | **Congruence × LR × AP** | 1 | 40 | 1.17 | 0.29 | 0.03 | |
| | **Congruence × LR × AP × Group** | 1 | 40 | 0.48 | 0.49 | 0.01 | |
| | **Emotion × Congruence × LR × AP** | 1 | 40 | 1.56 | 0.22 | 0.04 | |
| | **Emotion × Congruence × LR × AP × Group** | 1 | 40 | 0.71 | 0.41 | 0.02 | |
| **Midline** | **Group** | 1 | 40 | 1.19 | 0.28 | 0.03 | |
| | **Emotion** | 1 | 40 | 0.08 | 0.77 | 0.00 | |
| | **Emotion × Group** | 1 | 40 | 0.39 | 0.54 | 0.01 | |
| | **Congruence** | 1 | 40 | 2.24 | 0.14 | 0.05 | |
| | **Congruence × Group** | 1 | 40 | 0.07 | 0.80 | 0.00 | |
| | **AP** | 1 | 40 | 13.28 | <0.01 | 0.25 | * |
| | **AP× Group** | 1 | 40 | 1.10 | 0.30 | 0.03 | |
| | **Emotion × Congruence** | 1 | 40 | 0.62 | 0.44 | 0.02 | |

| | | | | | | |
|---|---|---|---|---|---|---|
| | **Emotion × Congruence × Group** | 1 | 40 | 0.08 | 0.78 | 0.00 |
| | **Emotion × AP** | 1 | 40 | 0.27 | 0.60 | 0.01 |
| | **Emotion × AP × Group** | 1 | 40 | 0.80 | 0.38 | 0.02 |
| | **Congruence × AP** | 1 | 40 | 1.03 | 0.32 | 0.03 |
| | **Congruence × AP × Group** | 1 | 40 | 0.06 | 0.80 | 0.00 |
| | **Emotion × Congruence × AP** | 1 | 40 | 0.89 | 0.35 | 0.02 |
| | **Emotion × Congruence × AP × Group** | 1 | 40 | 1.31 | 0.26 | 0.03 |
