# Supplementary material for: Intonation processing deficits of emotional words among Mandarin Chinese speakers with congenital amusia: an ERP study
Source: Front Psychol. 2015 Apr 9;6:385. doi: 10.3389/fpsyg.2015.00385 (PMC4391227; doi:10.3389/fpsyg.2015.00385)
Supplement: Supplementary file 2 [file Table2.PDF]

Supplementary Material

Intonation Processing Deficits of Emotional Words among Mandarin Chinese Speakers with Congenital Amusia: An ERP Study

Xuejing Lu [1,2], Hao Tam Ho[1], Fang Liu[3], Daxing Wu[2]*, William F. Thompson[1]*

[1] Department of Psychology, Macquarie University, Sydney, NSW, Australia
[2] Medical Psychological Institute, the Second Xiangya Hospital, Central South University, Changsha, China
[3] Department of Speech, Hearing and Phonetic Sciences, University College London, London, UK

*** Correspondence:**

Daxing Wu, Medical Psychological Institute, the Second Xiangya Hospital, Central South University, No.139 Middle Renmin Road, Changsha, 410011, China.
wudaxing2012@126.com

William F. Thompson, Department of Psychology, Macquarie University, NSW 2109, Australia
bill.thompson@mq.edu.au

Supplementary Tables

Supplementary Table 2. Statistical results for the N2 (250 - 320 msec) time window. Summary of repeated-measures ANOVAs on the mean amplitudes computed across the four regions of interest (ROI) and midline electrode sites, with the factors of Group (control / amusic), Emotion (positive / negative), Congruence (congruent / incongruent), LR (left / right) and AP (anterior / posterior). Effect size was estimated using partial eta-squared (η^2). DF refers to the degrees of freedom and the asterisks (*) indicate effects that yielded a significance level of $p < 0.05$.

	Effect	DF*n*	DF*d*	F	p	η^2	p<0.05
**ROI**	**Group**	1	40	6.35	0.02	0.14	*
	**Emotion**	1	40	17.63	< 0.01	0.31	*
	**Emotion × Group**	1	40	0.34	0.56	0.01	
	**Congruence**	1	40	1.40	0.24	0.03	
	**Congruence × Group**	1	40	3.87	0.06	0.09	
	**LR**	1	40	0.23	0.63	0.01	
	**LR × Group**	1	40	12.38	< 0.01	0.24	*

	**AP**	1	40	20.06	< 0.01	0.34	*
	**AP × Group**	1	40	3.30	0.08	0.08	
	**Emotion × Congruence**	1	40	0.37	0.55	0.01	
	**Emotion × Congruence × Group**	1	40	0.27	0.61	0.01	
	**Emotion × LR**	1	40	2.23	0.14	0.05	
	**Emotion × LR × Group**	1	40	2.19	0.15	0.05	
	**Congruence × LR**	1	40	0.00	0.98	0.00	
	**Congruence × LR × Group**	1	40	0.06	0.81	0.00	
	**Emotion × Congruence × LR**	1	40	0.09	0.77	0.00	
	**Emotion × Congruence× LR × Group**	1	40	0.02	0.88	0.00	
	**Emotion × AP**	1	40	3.64	0.06	0.08	
	**Emotion × AP × Group**	1	40	0.12	0.73	0.00	
	**Congruence × AP**	1	40	0.79	0.38	0.02	
	**Congruence × AP × Group**	1	40	0.32	0.58	0.01	
	**Emotion × Congruence × AP**	1	40	0.38	0.54	0.01	
	**Emotion × Congruence × AP × Group**	1	40	0.72	0.40	0.02	
	**LR × AP**	1	40	3.94	0.05	0.09	
	**LR × AP × Group**	1	40	2.04	0.16	0.05	
	**Emotion × LR × AP**	1	40	2.11	0.16	0.05	
	**Emotion × LR × AP × Group**	1	40	3.50	0.07	0.08	
	**Congruence× LR × AP**	1	40	0.09	0.77	0.00	
	**Congruence × LR × AP × Group**	1	40	0.02	0.89	0.00	
	**Emotion × Congruence × LR × AP**	1	40	1.09	0.30	0.03	
	**Emotion × Congruence × LR × AP × Group**	1	40	0.05	0.82	0.00	
**Midline**	**Group**	1	40	6.43	0.02	0.14	*
	**Emotion**	1	40	11.60	< 0.01	0.23	*
	**Emotion × Group**	1	40	0.53	0.47	0.01	
	**Congruence**	1	40	1.10	0.30	0.03	
	**Congruence × Group**	1	40	1.66	0.21	0.04	

**AP**	1	40	37.42	< 0.01	0.48	*
**AP × Group**	1	40	0.84	0.37	0.02	
**Emotion × Congruence**	1	40	0.07	0.79	0.00	
**Emotion × Congruence × Group**	1	40	0.06	0.82	0.00	
**Emotion × AP**	1	40	9.58	< 0.01	0.19	*
**Emotion × AP × Group**	1	40	0.08	0.79	0.00	
**Congruence× AP**	1	40	0.98	0.33	0.02	
**Congruence× AP × Group**	1	40	0.33	0.57	0.01	
**Emotion × Congruence × AP**	1	40	0.44	0.51	0.01	
**Emotion × Congruence × AP × Group**	1	40	1.53	0.22	0.04	
