# Supplementary material for: Intonation processing deficits of emotional words among Mandarin Chinese speakers with congenital amusia: an ERP study
Source: Front Psychol. 2015 Apr 9;6:385. doi: 10.3389/fpsyg.2015.00385 (PMC4391227; doi:10.3389/fpsyg.2015.00385)
Supplement: Supplementary file 3 [file Table3.PDF]

Supplementary Material

Intonation Processing Deficits of Emotional words among Mandarin Chinese Speakers with Congenital Amusia: An ERP Study

Xuejing Lu [1,2], Hao Tam Ho[1], Fang Liu[3], Daxing Wu[2]*, William F. Thompson[1]*

[1] Department of Psychology, Macquarie University, Sydney, NSW, Australia
[2] Medical Psychological Institute, the Second Xiangya Hospital, Central South University, Changsha, China
[3] Department of Speech, Hearing and Phonetic Sciences, University College London, London, UK

*** Correspondence:**

Daxing Wu, Medical Psychological Institute, the Second Xiangya Hospital, Central South University, No.139 Middle Renmin Road, Changsha, 410011, China.
wudaxing2012@126.com

William F. Thompson, Department of Psychology, Macquarie University, NSW 2109, Australia
bill.thompson@mq.edu.au

Supplementary Tables

Supplementary Table 3. Correlation results among musical skill (MBEA scores), language ability (behavioural task performance), and brain activity (mean amplitude for congruent and incongruent condition cross all ROI electrodes) within in N1 time window (3A) and N2 time window (3B). Summary of the Pearson correlation (2-tailed) were listed. * referred to a significant correlation at 0.05 level.

(A) N1 time window

**Group**		**MBEA**	**Task performance**	**Congruent**	**Incongruent**
**Amusics**	**MBEA**	1			
	**Task performance**	-0.12	1		
	**Congruent**	0.09	0.22	1	
	**Incongruent**	0.26	-0.25	0.18	1
**Controls**	**MBEA**	1			
	**Task performance**	0.11	1		

	**Congruent**	0.08	0.05	1	
	**Incongruent**	-0.31	-0.07	0.49*	1

(B) N2 time window

Group		MBEA	Task performance	Congruent	Incongruent
**Amusics**	**MBEA**	1			
	**Task performance**	-0.12	1		
	**Congruent**	0.19	-0.18	1	
	**Incongruent**	-0.14	-0.23	0.47	1
**Controls**	**MBEA**	1			
	**Task performance**	0.11	1		
	**Congruent**	-0.24	-0.05	1	
	**Incongruent**	0.48*	-0.15	-0.27	1
